# Supplementary material for: Fatal Epileptic Seizures in Mice Having Compromised Glutathione and Ascorbic Acid Biosynthesis
Source: Antioxidants (Basel). 2023 Feb 10;12(2):448. doi: 10.3390/antiox12020448 (PMC9952205; doi:10.3390/antiox12020448)
Supplement: Supplementary file 1 [file antioxidants-12-00448-s001.zip › antioxidants-2180981-supplementary.pdf]

Fatal Epileptic Seizures in Mice Having Compromised Glutathione and Ascorbic Acid Biosynthesis

Ying Chen[1,2#], Katherine D. Holland[3,4], Howard G. Shertzer[1], Daniel W. Nebert[1,5] and Timothy P. Dalton[1]

[1]Department of Environmental and Public Health Sciences and Center for Environmental Genetics, University of Cincinnati College of Medicine, Cincinnati, OH, 45267, USA.
[2]Department of Environmental Health Sciences, Yale School of Public Health, Yale University, New Haven, CT 06520, USA.
[3]Division of Neurology, Cincinnati Children's Hospital Medical Center, Cincinnati, OH, 45229, USA.
[4]Department of Pediatrics, University of Cincinnati College of Medicine, Cincinnati, OH, 45267, USA.
[5]Departments of Pediatrics and Molecular & Developmental Biology, Cincinnati Children's Research Center, Cincinnati, OH, 45229, USA.

[#]Corresponding Author: Ying Chen, PhD, Department of Environmental Health Sciences, Yale School of Public Health, 60 College Street, Rm. 521, New Haven, CT 06520-8034. Tel: 203.785.4694; Fax: 203.724.6023; Email: ying.chen@yale.edu.

Materials and Methods

Genotyping by polymerase chain reaction (PCR)

Genotypes of experimental mice were determined by PCR analysis of genomic DNA extracted from pup ear punches at PND12. Briefly, a 2 mm ear punch was lysed in 100 ul DirectPCR Lysis Reagent (Viagen Biotech, Los Angeles, CA) containing proteinase K (Sigma-Aldrich, St. Louis, MO) at 55°C overnight and denatured at 85°C for 45 min. 1 ul of crude DNA was used in two separate multiplex PCR reactions to detect *Gclm* and *Gulo* alleles, respectively, as described previously [1; 2].

Reverse transcription and real-time quantitative PCR (Q-PCR)

Total RNA was isolated from frozen liver pieces using RNeasy Plus Kit (Qiagen, Germantown, MD) according to manufacturer's protocol. cDNA was synthesized using iScript™ cDNA Synthesis Kit (BioRad, Hercules, CA) according to manufacturer's instructions using total RNA (1 μg from neocortex and 0.5 μg from hippocampus) in a 20 μl reaction volume. Q-PCR reaction mixtures contained 1 μl cDNA, 1x SYBR Green Supermix (BioRad), and 0.15 μM gene-specific primer sets in a total volume of 10 μl. Reactions were run using the CFX96 Touch Detection System (BioRad, Hercules, CA). Expression of housekeeping genes (*Rplp0* and *18s*) were used for normalization of C_T data using the delta-C_T method ($dC_T = C_T$(gene of interest) – C_T(average of housekeeping genes)), such that a high dC_T value was reflective of a low mRNA expression. Sequences of primers used for Q-PCR were 5′-GTAACCCGTTGAACCCCATT (forward) and 5′-CCATCCAATCGGTAGTAGCG (reverse) for *18s*, 5′-TGAGATTCGGGATATGCTGTTGG (forward) and 5′-CGGGTCCTAGACCAGTGTTCT (reverse) for *Rplp0*, 5′-ACCGGGAACCTGCTCAACT (forward) and 5′-GCATGGGACATGGTGCATTCC (reverse) for *Gclm*, 5′-GGCGATGTTCTTGAGACTCTGC (forward) and 5′-TTCCTTCGATCATGTAACTCCCATA (reverse) for *Gclc*, 5′-CTTGCGTGAATGTTGGATGTG (forward) and 5′-GCATCCCTTTTCTGCTTGATG (reverse) for *Gsr*, 5′-ATGAAAGTCCAGCCCAAGG (forward) and 5′-GGTCCTTCTCTATCACCTGGG (reverse) for *Gpx4*, 5′-CCGGCAGCAGAACAAGAAAGTGAA (forward) and 5′-AGGGCCAGGCCATGCTTGT (reverse) for *Gulo*, 5′-TTCGGCTTGCTCCATTCTCCTGAT (forward) and 5′-ATCAGTTCGAGCCTGGAAGCCATA (reverse) for *Slc23a1*, and 5′-GGACGGCATACAAGTTCCAGCTTT (forward) and 5′-CCCACTGAAATGGGTATGGAACCT (reverse) for *Slc23a2*.

Supplemental data

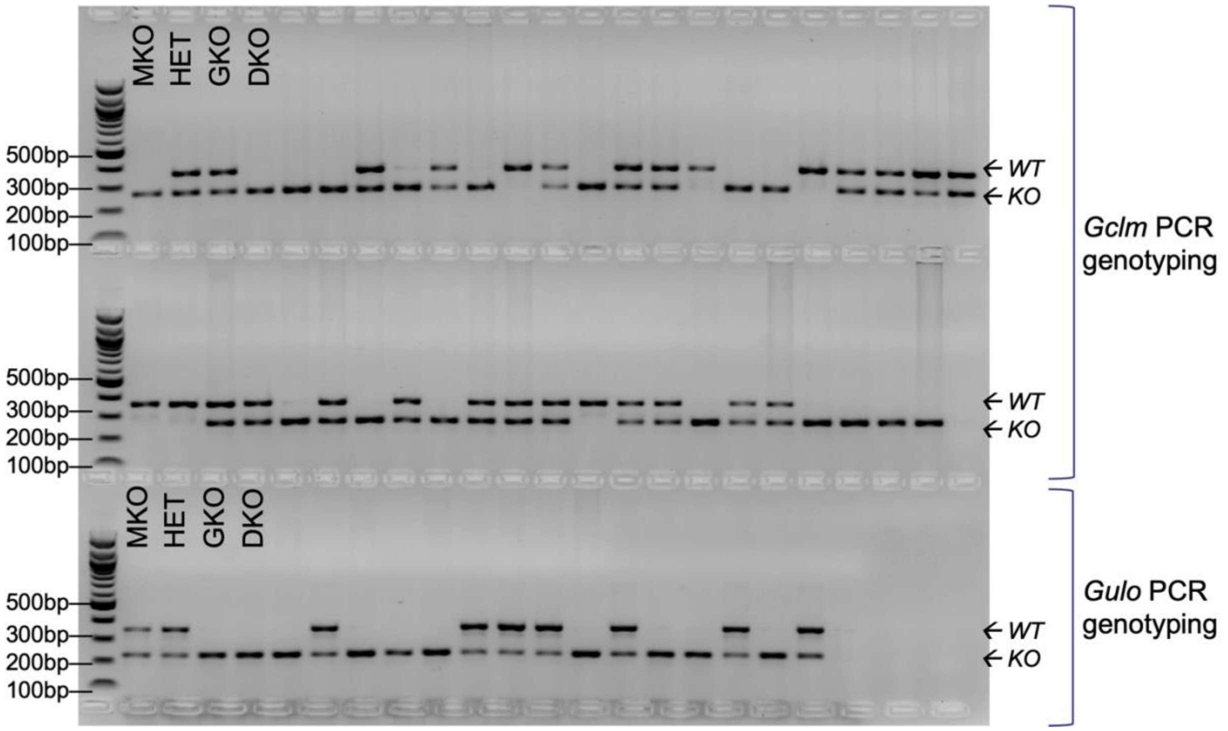

Figure S1. **Genotyping of experimental mice by PCR.** DNA electrophoresis of PCR genotyping using genomic DNA extracted from ear punches at PND12. *Gclm* WT and KO alleles yielded a 350 bp and 250 bp PCR products, respectively. *Gulo* WT and KO alleles yielded a 330 bp and 210 bp PCR products, respectively. Four genotype groups were $Gclm^{WT/KO}/Gulo^{WT/KO}$ (HET), $Gclm^{KO/KO}/Gulo^{WT/KO}$ (MKO), $Gclm^{WT/KO}/Gulo^{KO/KO}$ (GKO) and $Gclm^{KO/KO}/Gulo^{KO/KO}$ (DKO).

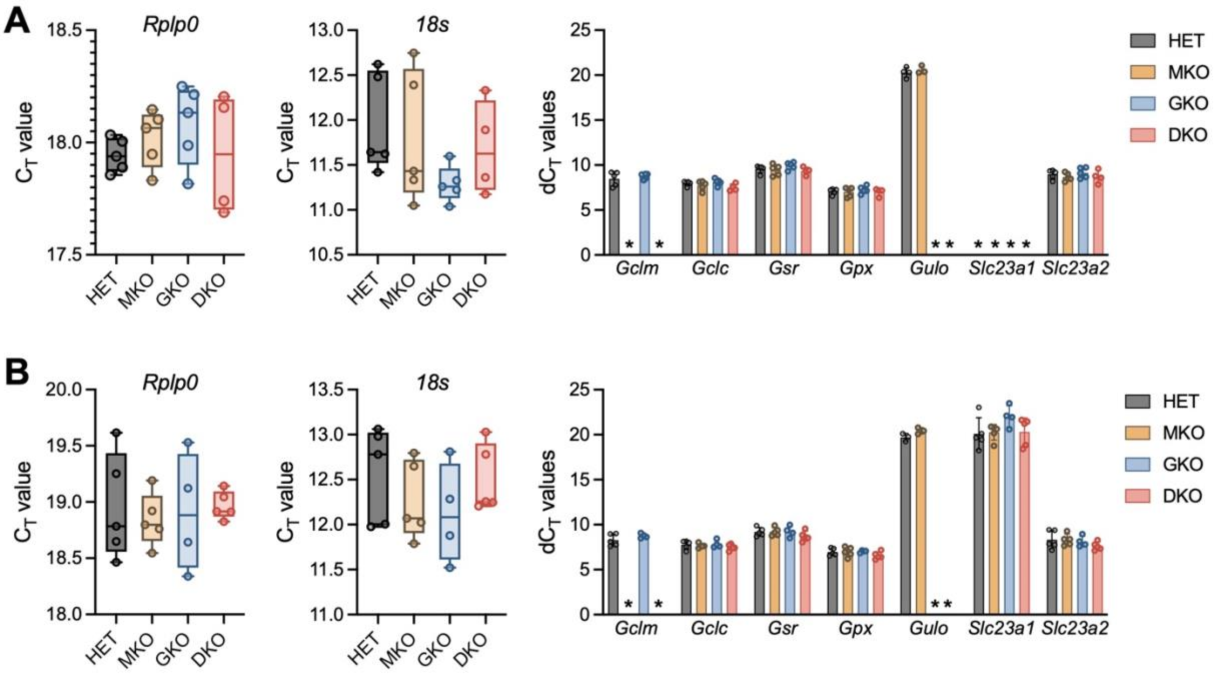

Figure S2. Q-PCR analysis of gene expression in brain tissues. Examined genes were GSH metabolizing enzymes (*Gclm, Gclc, Gsr*, and *Gpx*), AA biosynthesis enzyme (*Gulo*), and AA transporters (*Slc23a1* and *Slc23a2*) in neocortex (***A***) and hippocampus (***B***). Expressions of housekeeping genes *Rplp0* and *18s* were used for normalization of C_T data. Relative mRNA abundance of individual gene was reported as the dC_T value wherein a high dC_T value was reflective of a low mRNA expression. Multiple group comparisons were performed by one-way ANOVA followed by *post-hoc* Bonferroni test for multiple comparisons using the Graphpad Prism software (San Diego, CA). $P < 0.05$ was considered significant. *mRNA of the gene was not detectable in the specified genotype group. Our data showed that: (i) As expected, the expression of *Gclm* gene was not detectable in MKO or DKO mice; likewise, the expression of *Gulo* gene was not detectable in GKO or DKO mice. (ii) The *Gulo* and *Slc23a1* genes were expressed at minimal levels in these brain tissues. (iii) There was no difference in the expression of these genes in the two brain regions among four genotype groups.

References

[1] Y. Yang, M.Z. Dieter, Y. Chen, H.G. Shertzer, D.W. Nebert, and T.P. Dalton, Initial characterization of the glutamate-cysteine ligase modifier subunit Gclm(-/-) knockout mouse. Novel model system for a severely compromised oxidative stress response. Journal of Biological Chemistry 277 (2002) 49446-52.

[2] N. Maeda, H. Hagihara, Y. Nakata, S. Hiller, J. Wilder, and R. Reddick, Aortic wall damage in mice unable to synthesize ascorbic acid. Proc Natl Acad Sci U S A 97 (2000) 841-6.
